# Economic evaluations of ergonomic interventions preventing work-related musculoskeletal disorders: a systematic review of organizational-level interventions

**DOI:** 10.1186/s12889-017-4935-y

**Published:** 2017-12-08

**Authors:** Hélène Sultan-Taïeb, Annick Parent-Lamarche, Aurélie Gaillard, Susan Stock, Nektaria Nicolakakis, Quan Nha Hong, Michel Vezina, Youssouph Coulibaly, Nicole Vézina, Diane Berthelette

**Affiliations:** 1 École des Sciences de la Gestion, UQÀM - Université du Québec à Montréal, 315, rue Sainte-Catherine Est, Montréal, Québec H2X 1L7 Canada; 2 Université du Québec à Trois-Rivières, 3351, boul. des Forges, C.P. 500, Trois-Rivières, Québec G9A 5H7 Canada; 3 LEDI, Pôle d’économie et de gestion, Université de Bourgogne, 2 boulevard Gabriel, BP 26611, 21066 Dijon cedex, France; 4 Institut National de Santé Publique du Québec and School of Public Health, Université de Montréal, 190 boulevard Crémazie est, Montréal, Québec H2P 1E2 Canada; 5 McGill University, 5858, Chemin de la Côte-des-Neiges, Suite 300, Montreal, QC H3S 1Z1 Canada; 6 Institut National de Santé Publique du Québec, 945 avenue Wolfe, Québec, G1V 5B3 Canada; 7 UQAM- Université du Québec à Montréal, 141 avenue du Président Kennedy, Montréal, Québec H2X 1Y4 Canada

**Keywords:** Work-related musculoskeletal disorders, Occupation, Prevention, Systematic review, Mixed methods, Ergonomics, Cost-benefit, Cost-effectiveness, Economic evaluation, Intervention

## Abstract

**Background:**

Work-related musculoskeletal disorders (WMSD) represent a major public health problem and economic burden to employers, workers and health insurance systems. This systematic review had two objectives: (1) to analyze the cost-benefit results of organizational-level ergonomic workplace-based interventions aimed at preventing WMSD, (2) to explore factors related to the implementation process of these interventions (obstacles and facilitating factors) in order to identify whether economic results may be due to a successful or unsuccessful implementation.

**Methods:**

Systematic review. Studies were searched in eight electronic databases and in reference lists of included studies. Companion papers were identified through backward and forward citation tracking. A quality assessment tool was developed following guidelines available in the literature. An integration of quantitative economic results and qualitative implementation data was conducted following an explanatory sequential design.

**Results:**

Out of 189 records, nine studies met selection criteria and were included in our review. Out of nine included studies, grouped into four types of interventions, seven yielded positive economic results, one produced a negative result and one mixed results (negative cost-effectiveness and positive net benefit). However, the level of evidence was limited for the four types of interventions given the quality and the limited number of studies identified. Our review shows that among the nine included studies, negative and mixed economic results were observed when the dose delivered and received by participants was low, when the support from top and/or middle management was limited either due to limited participation of supervisors in training sessions or a lack of financial resources and when adequacy of intervention to workers’ needs was low. In studies where economic results were positive, implementation data showed strong support from supervisors and a high rate of employee participation.

**Conclusion:**

Studies investigating the determinants of financial outcomes of prevention related to implementation process are very seldom. We recommend that in future research economic evaluation should include information on the implementation process in order to permit the interpretation of economic results and enhance the generalizability of results. This is also necessary for knowledge transfer and utilization of research results for prevention-oriented decision-making in occupational health and safety.

**Electronic supplementary material:**

The online version of this article (10.1186/s12889-017-4935-y) contains supplementary material, which is available to authorized users.

## Background

Work-related musculoskeletal disorders (WMSD) represent a major public health problem and economic burden to employers, workers and health insurance systems. In the United States between 1992 and 2010, WMSD accounted for 29–35% of all occupational injuries and illnesses and accounted for a large number of work days lost as compared to other occupational illnesses [1]. According to the results of a provincial survey conducted in 2007–2008, one in five workers in the province of Québec, Canada, experienced non-traumatic WMSD in at least one body region in the 12 months prior to the survey [2]. In France, non-traumatic WMSD are the first cause of compensated occupational diseases and have been increasing at an annual rate of approximately 18% over the last 10 years. Workers’ compensation claims due to musculoskeletal disorders represented 76% of total occupational disease-related claims in France in 2015 [3].

WMSD refer to non-traumatic inflammatory or degenerative disorders of the musculoskeletal structures of the neck, back, upper or lower extremities. They develop over time and arise when the adaptive and repair capacities of affected structures have been exceeded [4]. The role of physical and psychosocial work exposures in the development of WMSD is now well established [5–8]. We also know that there is modest evidence that some ergonomic interventions may reduce these occupational exposures and have a significant effect on WMSD incidence or prevalence among workers [9–12].

However, little is known about the cost-benefit or cost-effectiveness ratios of workplace-based ergonomic interventions aimed at preventing WMSD and very few literature reviews are available. One review focused solely on return-to-work interventions among employees with back pain [13]. Two reviews analyzed evaluation studies of ergonomic interventions with a focus on economic outcome measures from the employer’s perspective (gains): one of these included outcome measures which could be converted into avoided monetary costs [14], while the other emphasized the effects of ergonomic interventions on product quality [15]. However, the lack of information on the costs of interventions did not allow cost-benefit approaches. Although the systematic review by Tompa et al. [16] provided very valuable insights into the results of economic evaluations of ergonomic interventions, it is the most recent review of this type and several studies on ergonomic interventions have been published since then.

Such economic results provide very useful information for decision-making in the field of occupational health and safety (OHS). In particular, detailed information on the financial implications of ergonomic interventions (costs of intervention and gains provided by intervention) can provide valuable inputs into the way decision-makers deal with OHS issues. Enterprise decision-making in OHS is based on an initial trade-off between undertaking or not preventive actions, and a second trade-off between various alternative preventive interventions corresponding to different levels of investment in OHS. Economic evaluations provide relevant decision-making tools for the allocation of scarce resources for prevention [17, 18].

This systematic review has two main objectives: (1) to analyze the cost-benefit results of workplace-based interventions with an organizational dimension aimed at preventing WMSD and (2) to explore factors related to the implementation process of these interventions in order to identify whether economic results may be due to a successful or unsuccessful implementation (also called program failure) [19]. This review is part of a larger project conducted for the Québec Public Health Network in Occupational Health with developing ergonomic intervention strategies and testing their implementation in various workplaces in order to prevent WMSD. Given this context, this review was designed as an evidence-based tool for decision-making regarding the choice of cost-beneficial interventions. To make it possible to interpret the economic results produced by intervention studies, we analyzed published companion papers relating to the intervention (protocol design, effectiveness evaluation, implementation studies on the intervention). This integration of quantitative economic results and qualitative implementation data in a mixed methods approach is very rare in the literature and constitutes an innovative feature of the current study.

We focused on workplace-based interventions that are not solely applied at the individual level (such as workstation accommodation) but also intend to bring about changes at the organizational level. The organizational environment has been identified as a determinant of the onset of non-traumatic WMSD [7, 8]. It might also be more difficult and more costly to implement changes at the organizational level, thus making cost-benefit analyses of this type of intervention all the more relevant. To our knowledge, this is the first systematic review on the cost-benefit of workplace-based interventions with an organizational dimension.

## Methods

### Search strategy

Studies were searched in the following eight electronic databases: MEDLINE, PreMEDLINE, EMBASE, CINAHL, PsycINFO, Business Source Complete, EconLit and EBMR-NHSEED. These databases were chosen to extensively cover the scope of this systematic review and include health, management, economic as well as OHS references. The results of each database search were transferred to EndNote (bibliographic management software) and duplicates were removed.

A comprehensive set of search terms was developed (copy available from authors) in order to combine four generic concepts: workers/occupational health (as target population), prevention intervention at an organizational level (as action), WMSD (as outcome), economic evaluation (as evaluation type). A librarian specializing in bibliographic searches in the OHS field helped develop the search strategy syntax for each database. We used multiple combinations of natural language in the Title and Abstract fields and also subject heading or descriptors (controlled vocabulary) in database thesaurus. For EBMR-NHSEED, Business Source Complete and Econlit, we used the combination of three concepts instead of four because adding the concept of “prevention intervention with organizational dimension” made the search too restrictive. The search was limited to articles in English or French published between January 1 2000 and November 24 2015.

Two additional sources of information were used to find relevant studies. First, from the systematic database searches, seven literature review papers including economic evaluation studies were found. The articles cited in the reference list of these seven reviews were screened. Second, the reference lists of the included studies were scrutinized for additional studies.

In addition, companion papers were identified by using references in the included economic studies (backward citation tracking), and by searching for articles in Google Scholar and Medline using names of researchers who authored the included economic studies (forward citation tracking).

### Screening and selection criteria

Only studies published in peer-reviewed journals were considered. Two researchers (HST, APL) independently screened all titles and abstracts. A second screening was conducted by the same researchers based on full texts.

The following selection criteria were applied.

Inclusion criteria:

1. Interventions aimed at preventing work-related musculoskeletal pain or disorders in the neck, back, shoulders, elbows, forearms, wrists or hands and/or lower limbs;
2. Economic evaluations: Studies had to provide a cost-efficiency, cost-utility or cost-benefit analysis [20]. We also included payback period estimates and return-on-investment studies;
3. Workplace-based interventions at an organizational level, following Montano et al.’s classification [21]: interventions that modified work time-related conditions (such as job rotation, work pace), processes and procedures (task distribution, design and conception of training at the organizational level) were included. Interventions were also included when workplace psychosocial factors were modified, for instance when supervisors were involved in providing feedback, in participating in problem identification and in developing solutions. As a result, participatory ergonomic approaches involving a worker risk assessment team and contribution from supervisors were also included;
4. Written in English or French;
5. Published between 2000 and 2015.

Exclusion criteria:

1. Studies that did not evaluate outcomes specifically focused on musculoskeletal symptoms or disorders;
2. Return-to-work and rehabilitation interventions;
3. Interventions aimed at recruitment screening of future workers;
4. Interventions implemented outside the workplace, such as in a clinical or medical setting;
5. Synthesis or point-of-view studies which did not evaluate specific interventions;
6. Partial economic evaluations with data on the costs of intervention only, or data on the benefits only;
7. Interventions without an organizational dimension, such as individual workstation adjustment with training limited to individual instruction in the use of workstation equipment;
8. Training in coping strategies implemented at the individual level only, without examining work organization (e.g., change in work pace, job rotation, supervision), and without supervisors collaboration or involvement. For example, interventions relating to lifting equipment were not included if the intervention did not include a written zero-lift policy, or a no unsafe-manual lift policy at the organizational level.

Given the low number of studies identified in the literature meeting both the subject relevance (inclusion criteria 1, 2) and the organizational level criterion (inclusion criterion 3), we did not apply inclusion or exclusion criteria based on study design. The only selection criteria related to study design were the presence of measures of costs and benefits of the intervention in the study (inclusion criterion 2 and exclusion criterion 6). Study design was nonetheless taken into account in the evaluation of the methodological quality of each study.

In addition, to interpret the economic results produced by the included studies, we identified all companion papers published in connection with the intervention that contained an analysis of the same intervention as that dealt with in the principal economic evaluation paper, i.e. implemented in the same company and at the same time. The principal paper was the one that included the most detailed economic evaluation of the intervention.

### Quality assessment

All studies that met both the subject relevance and organizational criteria passed through a quality assessment process. We developed a quality assessment tool based on criteria published in recent literature reviews and guidelines for the evaluation of economic studies [16, 20, 22–25]. This assessment tool was validated following consultation with a scientific team composed of experts in economic evaluation and WMSD intervention (HST, SS, MV, DB) and is presented in Table 1. It includes 18 criteria subdivided into three broad categories: 1) Purpose of the study and nature of the intervention, 2) Study design and evaluation of intervention effectiveness, and 3) Features specific to the economic evaluation. Each item was ranked on a three-point scale (0, 1, 2), except for the last criterion (*Were costs and outcomes that occur in the future discounted to their present value?*). This criterion was scored on a 2-point scale (0, 1) since discounting or not was debatable given the limited duration of follow-up in the included studies. To achieve a perfect score (100%), studies had to obtain 35 points from 18 criteria.

**Table 1:** Quality assessment tool and average score obtained for each criterion by the included 9 studies

Purpose of the study and nature of intervention	Average score for each criterion
1. Were the objective and economic perspective of the evaluation clearly and explicitly stated?	2.0 /2
2. Were workers’ exposure to intervention and involvement into intervention documented and appropriate?	0.9 /2
3. Were changes implemented as intended?	1.1 /2
Study design and evaluation of intervention effectiveness	
4. Did the study include a control group?	0.6 /2
5. Were study participants randomly assigned to the control or intervention groups? If study participants were not randomly assigned, were workers’ baseline characteristics measured?	0.9 /2
6. Were outcome indicators measured before and after the intervention?	2.0 /2
7. Were contextual factors and co-interventions that could influence the results taken into account in the analysis or in the interpretation of the results?	0.8 /2
8. Was the statistical analysis appropriate for measuring the effectiveness of the intervention?	1.1 /2
9. Were study participants data paired before and after intervention?	0.8 /2
10. Was the length of follow-up after the end of implementation of the intervention appropriate or justified by the authors?	1.4 /2
Features specific to economic evaluation	
11. Did the study involve a comparison of competing alternatives and was there a comprehensive description of these alternatives?	0.8 /2
12. Were all important and relevant costs and outcomes for each alternative identified and measured in appropriate physical units, given the evaluation perspective?	1.7 /2
13. Was the method used for cost assessment explicitly stated and justified?	1.4 /2
14. Was an incremental analysis of costs and outcomes of alternatives performed?	0.8 /2
15. Were all important variables, whose values are uncertain, appropriately subjected to sensitivity analysis or presented with confidence intervals?	0.6 /2
16. Did the presentation and discussion of study results include all issues of concern?	1.4 /2
17. Did the study discuss the generalizability of the results to other settings and populations?	0.2 /2
18. Were costs and outcomes that occur in the future discounted to their present value?	0.2 /2

*Average score calculated as the average of the scores obtained by the 9 included studies for each criterion (if all studies get a score of 2 for criterion 1, then the average score of this criterion is 2). Studies could get a score of 0 or 1 for criterion 18. The average score for this criterion was multiplied by 2 in* Table 1 *to be comparable to the other criteria*

Two researchers (HST, AG, both economists with a background in OHS) scored each study independently and then met to reach a consensus when differences were observed.

### Data extraction tool for economic evaluation data (Additional file 1: Table S1)

A tool to extract quantitative economic data was developed based on the frameworks used in published systematic reviews of economic evaluations of preventive interventions in OHS [16, 22, 26]. The tool was designed in order to be comprehensive and include all categories of economic data considered as relevant in these published reviews. Discussions among the co-researchers of this article resulted in some categories being excluded from the data extraction tool but included in the quality assessment tool (for example statistical analysis for uncertainty assessment). As a result, the data extraction tool for economic evaluation included the following elements:

1. Methods of the economic evaluation: design, population, sample size, type of evaluation, economic perspective of the evaluation,
2. Characteristics of the economic evaluation: indicators for outcomes and intervention costs, statistical analysis, results.

Two researchers (HST, AG) independently extracted economic data from all studies and then met to reach a consensus when differences were observed.

### Data extraction tool for implementation process data (Additional file 1: Table S2)

A tool to extract data on the implementation process from the main economic evaluation study and its companion papers was developed based on the Linnan and Steckler framework [27], adapted by Saunders et al. [28] and widely used in the literature [29]. In our data extraction tool, all seven components of Saunders’s framework have been included (fidelity, dose delivered, dose received, satisfaction, reach of targeted population, participation, context). We completed this framework with an additional component related to workers’ needs assessment and adequacy of intervention to workers’ needs, which is emphasized as a key element of the implementation process in the literature [30–32]. Moreover, for each factor we documented whether it was presented in the article as an element in favour of the success of the intervention (indicated with a “+”) or an element contributing to the failure of the intervention (indicated with a “-”). Our data extraction tool documents the following elements:

1. description of the intervention and treatment of the control group,
2. assessment of workers’ needs (diagnosis) before implementation and adequacy of intervention to workers’ needs,
3. proportion of employees reached by the intervention, dose delivered and dose received, fidelity to intervention protocol,
4. contextual factors, co-interventions or other confounders that may have had an impact on outcomes measures,
5. obstacles and facilitators, authors’ hypotheses that may explain intervention effectiveness,
6. participants’ satisfaction toward intervention.

Three researchers (HST, APL, YC) extracted implementation data from all studies and then met to reach a consensus when differences were observed.

### Synthesis

We integrated the two sets of data (economic and implementation data) following an explanatory sequential design [33, 34]: in a first step, we synthesized the results of the economic evaluations and the quality assessment scores (quantitative data). In a second step, the findings from this first step were explained by the synthesis of implementation (qualitative) data. In mixed methods research, integration is “an intentional process by which the researcher brings quantitative and qualitative approaches together”, which “become interdependent in addressing common research questions” [35].

For the synthesis of the quantitative data, the studies were categorized based on the type of intervention. For each type of intervention, we described the economic results and rated the strength of evidence in order to produce a best-evidence synthesis of results available in the literature. There are numerous rating systems available in the literature, most of which take into account three criteria: the number of available studies, the quality of these studies (design, potential bias) and the consistency of results among the studies [36]. In this review, we used the criteria for levels of evidence elaborated by Tompa [16, 22] for literature reviews focusing on the same type of study as ours, i.e. economic evaluations of preventive interventions in OHS. Tompa’s approach derives from the best-evidence synthesis developed by Slavin [37] as an alternative to meta-analyses (Table 2).

**Table 2:** Criteria for levels of evidence

Level of evidence	Minimum criteria
Strong	3 high quality studies, agree on the same findings
Moderate	2 high quality studies agree or 1 high quality and 2 medium quality studies agree
Limited	1 high quality study or 2 medium quality studies agree
Mixed	Findings from medium and high quality studies are contradictory
Insufficient	No high quality study, only 1 medium quality study or any number of low quality studies

*Source: Adapted from Tompa* et al. *(2009, 2010)*

We then conducted a qualitative synthesis of the implementation data. We analyzed the determinants of the success or the failure of the intervention in the articles and synthesized this information for each category of intervention identified in the quantitative synthesis. This synthesis was performed by HST.

The integration between quantitative and qualitative data was intended to answer the following question: how might the quantitative results from the economic evaluation be explained by the implementation data? A comparative matrix of the implementation data, the economic outcomes and the level of evidence for each category of intervention was developed by HST and QNH as a result of this final synthesis (see Table 3).

## Results

### Literature searches

**Fig. 1:**
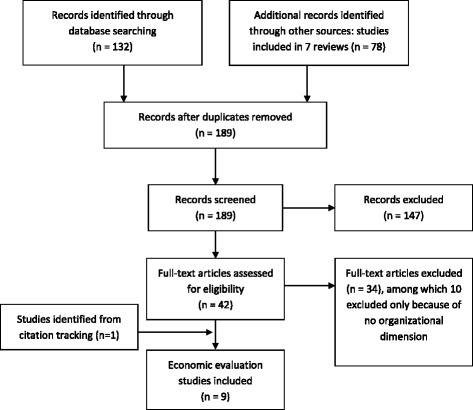
PRISMA Flow Diagram (Mohrer, 2009)

Out of 189 records, nine studies met selection criteria and were included in our review [38–46]. The results are shown in a PRISMA flow diagram (Fig. 1) [47].

Among excluded studies, 10 fulfilled all inclusion criteria except for the organizational dimension of intervention: they consisted in adaptations of the workstation and individual training [48–53], promotion of healthy life habits at an individual level [54, 55], a conference for employees on how to avoid back injury [56]. One article involving a three-case study did not provide enough information on the individual or organizational components of interventions [57].

The search for companion papers produced a total of 14 additional articles. Four studies did not have any available companion paper in the literature [39, 40, 42, 45], and the other five studies had from one to five companion papers available for the same intervention implemented in the same organization. These companion papers dealt with the protocol design, the measure of the effectiveness of the intervention, and only three analyzed the implementation process [58–60]. In two cases, the principal (economic) paper had another complementary economic article that complemented the analyses [61, 62].

### Main features of included studies

The studies covered a wide variety of industries and professional categories: computer workers, workers in installation companies, railway transportation workers, universities, airlines, steel company workers and construction workers. The only sector represented in more than one study was the healthcare sector, and this was indeed highly represented (five studies out of nine). Four studies were undertaken in the Netherlands, three in the United States, two in Canada (Additional file 1: Table S1).

The nine included studies could be categorized in four different types of interventions (detailed in Additional file 1: Table S2):

- Five studies evaluated interventions aimed to reduce lifting hazards associated with patient handling for healthcare workers through lift equipment for repositioning and transferring patients. These were accompanied by staff training and an organizational policy geared toward risk reduction (e.g., written no-unsafe manual lift policy) [39, 42, 43, 45, 46].
- Two studies analyzed participatory ergonomic interventions in which workers were involved in analysis of work factors contributing to musculoskeletal problems, elaboration and selection of solutions, prototyping, and implementation [40, 41].
- One intervention was implemented for computer workers through a series of meetings between employees and supervisors in order to bring about changes in work style (body posture, breaks, workload, work stress) and in physical activity [38].
- One intervention took place at a construction site and was aimed at reducing the workload at the individual level and changing workers’ influence on work organization through empowerment sessions [44]. It consisted in a series of individual meetings with a physical therapist, a tool to promote rest-breaks, and a collective component with two empowerment sessions with workers and supervisors.

Regarding the economic perspective used in evaluations (Additional file 1: Table S1), all the studies included an analysis from the employer’s point of view, two studies also provided results from the point of view of an insurance institution (workers’ compensation board of British Columbia in Canada), and one study from the societal point of view.

All studies used a before-after design and data were paired before and after the intervention in six of the nine studies. Three studies were randomized-controlled trials and six used a quasi-experimental uncontrolled design: five did not conduct a comparison with an alternative strategy and their approach was not incremental, one had an incremental approach with a simulation of effects without intervention [46].

The type of economic evaluation carried out was quite varied: three studies conducted a cost-effectiveness analysis, two studies a cost-benefit analysis, six estimated a payback period and two calculated a return on investment rate (some studies reported several analyses in the same article). The outcome indicators considered for the purposes of economic evaluation were also very diverse: number and costs of sick days, workers’ injury compensation claims, health care costs, prevalence of musculoskeletal symptoms, physical and mental health. Presenteeism costs were estimated in one study [44] and the cost of days of modified work were considered in one study [43].

### Quality assessment

Applying our quality assessment tool to the nine included studies, we identified four high-quality studies with scores ranging from 63% to 89%. Five studies had a low to very low score (ranging from 29% to 43%) (see Additional file 1: Table S1, first column).

As shown in Table 1, three criteria of the 18 criteria were very often fulfilled by the nine included studies and received high average scores (average grade from 1.6 to 2, very close to the maximum grade of 2): criterion 1 (clear objectives and perspective of the evaluation), criterion 6 (outcome indicators measured before and after intervention) and criterion 12 (adequate measures of costs and consequences). By contrast, eight criteria were rarely fulfilled (average score below 0.8): criterion 4 (inclusion of a control group), criterion 7 (account of co-interventions and contextual factors), criterion 9 (participants’ data paired), criterion 11 (comparison between competing alternatives) and 14 (incremental analysis), criterion 15 (analysis of the level of uncertainty of variables), criterion 17 (discussion about generalizability of results) and criterion 18 (discounting of costs and gains).

### Synthesis of economic results and level of evidence

Of the five studies that evaluated a very similar intervention – lifting equipment and policy for patient handling in the healthcare sector -, all concluded that the savings cumulated after intervention were higher than the total investment, with payback periods ranging from 3 to 5 years from the employer’s perspective, and from 0.82 to 9 years from the workers’ compensation board perspective. In all studies, lifting equipment and policies significantly reduced injuries and workers’ compensation claims.

Among these five studies, only one was a high-quality study [46], and the other four studies were of low quality [39, 42, 43, 45] (Additional file 1: Table S1). If we apply our criteria for evidence synthesis (Table 2), these studies provide limited evidence of the economic benefits of these interventions.

Two studies evaluated participatory ergonomic interventions: one high-quality study [41] and one low-quality study [40]. Driessen et al. [41] concluded that the intervention was not cost-effective from the societal point of view and that the net benefit was negative from the employer’s perspective. In the de Jong and Vink’s study (2002), the total investment was rapidly paid-back after a one-year period. The evidence from these studies is therefore limited with non-convergent economic results for participatory ergonomic interventions.

Two other high-quality studies were included but were too different from each other to be considered as belonging to the same intervention category. Bernaards et al. [38] evaluated a physical activity and work style intervention (body posture, static workload, breaks, work stress) for computer workers with neck and upper limb symptoms. The authors concluded that only the work style intervention was cost-effective in reducing average pain and improving recovery in the neck and shoulders, which was not the case for the work style plus physical activity intervention. Oude Hengel et al..... [44] evaluated an intervention aimed at reducing workload and improving empowerment (workers’ influence on work) among construction workers. This study concluded that the intervention was not cost-effective but that the net benefit was positive given a decrease in absenteeism. Given the great differences in sectors, work activity and nature of interventions, the level of evidence is considered as limited for both interventions.

### Integration of quantitative and qualitative data

In this section, we provide an integration of economic evaluation results along with the qualitative information provided in the included studies and companion papers (see Table 3).

The five studies on lifting equipment and policy in the health care sector reported positive economic results in terms of payback period and return on investment from the employer’s and workers’ compensation board perspectives. Nelson et al’s study [43] and its companion paper [63] revealed a short payback period (3.75 years) as well as Collins et al’s study [39] (3 years). An analysis of qualitative data showed that this intervention seemed to respond better to workers’ needs to lift and transfer patients than to their needs to reposition them [42, 46, 61, 64]. Our synthesis showed a strong support for the program from nurses, supervisors and co-workers, and from patients. A participatory process in equipment implementation facilitated nurses’ compliance with the use of the lifting equipment: their input on the selection of equipment favored staff buy-in [39].

The participatory ergonomic intervention evaluated in the high-quality study by Driessen et al. [41] was not cost-effective and the net benefit was negative. An analysis of the companion papers shows that only a small proportion of identified ergonomic solutions was effectively implemented. Working groups prioritized 66 ergonomic measures, of which 34% were fully implemented. Only 26% of workers perceived the ergonomic measures as implemented [58, 65]. The implementation of prioritized ergonomic measures was hampered by a shortage of financial resources and lack of time and resources [66]. According to the authors, the working groups prioritized the simpler and less expensive measures, and this may explain the absence of effect. Moreover, the prevalence of low back pain and neck pain was low among workers at baseline and they probably did not feel the need to participate to data collection and intervention [65]. Another participatory ergonomic intervention evaluated by de Jong et al. [40] reported a very short payback period of less than a year. The implementation data showed a relatively high dose delivered and dose received and indicated a strong management commitment to and support for the intervention. However, a low direct participation of workers was observed as well as a limited acceptance of changes by employees in some units.

The work style (WS) intervention evaluated by Bernaards et al. [38], which consisted of six interactive group meetings on body posture, workload, breaks and work stress among computer workers, was cost-effective, whereas the combined work style and physical activity (WSPA) intervention was not. A synthesis of implementation process data showed that several features may explain why the WSPA intervention did not produce positive financial outcomes: intervention on physical activity was not perceived by workers as corresponding to their needs [67, 68]. Workers participation was lower in the WSPA group and the physical activity component was not effective in increasing physical activity among participants [67]. The authors questioned whether group meetings were a suitable tool for increasing physical activity among workers, as opposed to individual coaching [67]. By contrast, workers’ participation to WS intervention was higher.

One study in the construction sector by Oude Hengel et al. [44] evaluated an intervention consisting of recommendations from a physical therapist on how to reduce workload, as well as empowerment sessions in order to improve workers’ influence on work. An examination of the outcomes, such as prevalence of musculoskeletal symptoms, showed that the intervention was not effective and not cost-effective. However, the cost of intervention was lower in the intervention group than in the usual care group, which received conventional training sessions on physical workload and safety issues. This lower cost contributed to a positive net benefit per worker when considering absenteeism as an outcome for cost-benefit analysis (Additional file 1: Table S1). A synthesis of implementation data showed that the fidelity to intervention protocol was low, as well as the dose received by workers. The workers’ level of satisfaction with regard to the empowerment sessions was low because supervisors and management did not participate in these sessions. Construction workers found it difficult to follow recommendations on rest breaks, the tool for rest-breaks was rarely used by workers and never served as a basis for discussion with supervisors [59, 69]. The authors’ hypothesis was that this intervention might have represented a program failure [69].

Table 3 presents, for the four categories of interventions, the integration of economic evaluation results, level of evidence, and implementation factors that may have contributed to the success or the failure of the intervention and that may have determined financial outcomes.

**Table 3:** Synthesis, integration of quantitative and qualitative data for each type of intervention

Intervention type, Level of evidence for economic results	Cost-beneficial? (reference to economic article)	Factors in favour of success of intervention	Factors in favour of failure of intervention
Lifting equipment for patients Limited, convergent results (5 studies)	Yes [39, 42, 43, 45, 46]	• Strong support from nurses, supervisors, co-workers, and patients • Nurses’ participation to intervention process • High adequacy to worker’s needs for lifting and transferring tasks	• Low adequacy to workers’ needs for repositioning tasks • Some difficulties in applying procedures (resisting, heavy patients, procedural errors)
Participatory ergonomic intervention Limited, non convergent results (2 studies)	No [41]	• High satisfaction among steering groups members • High attendance to meeting of steering groups members	• Limited dose delivered and dose received of fully implemented ergonomic measures • Lack of financial and personal resources • Low adequacy to perceived workers’ needs • Low satisfaction among workers
	Yes [40]	• Strong management support for the program • High dose delivered and received	• Low direct participation of workers • Limited acceptance by employees in some units
Work style intervention and/or physical activity Limited (1 study)	Yes (Work style, WS) No (Work style and physical activity, WSPA) [38]	• High participation of workers to WS (attendance to meetings)	• Lower participation of workers to WSPA (attendance to meetings) • Low adequacy with workers’ needs for the physical activity component of the intervention (WSPA) • Group meeting may not be suitable for increasing physical activity (WSPA)
Workload and empowerment Limited (1 study)	No and yes No effects on health and symptoms but decrease in sickness days [44]	• High dose delivered (except for physical activity training).	• Low dose received • Low fidelity to protocol • Low workers’ satisfaction toward an intervention tool (rest-break tool) • Difficulties in applying procedures • Economic crisis climate, job insecurity • Low support and commitment of supervisors

## Discussion

### Main findings

This systematic review retained nine economic evaluation studies on four different types of workplace-based interventions with an organizational dimension designed to reduce or prevent WMSD. Out of nine included studies, seven yielded positive economic results, one produced a negative result and another mixed results (negative cost-effectiveness and positive net benefit). However, the level of evidence was limited for all four types of interventions given the quality of the studies and the small number of studies available for consideration. Our review provides additional information to determine whether economic results were due to specific obstacles or facilitating factors in the implementation process through mixed synthesis of economic evaluation articles and 14 companion papers.

### Implications for practice

Our results have implications for decision makers involved in making investment decisions for prevention. It underlines the importance of a strong support for the intervention program from co-workers, supervisors and top management to get positive economic results. It also emphasizes that the adequacy of intervention to workers’ needs and employees’ participation are favorable factors for positive financial outcomes. Moreover, negative and mixed economic results were observed when the dose delivered and received by participants was low and support from management was limited either due to limited participation of supervisors in training sessions or due to a lack of financial resources.

These results show that financial outcomes from prevention do not only depend on the type of intervention program but also on the implementation process of the intervention. These results are consistent with the literature on the determinants of *health* outcomes in OHS preventive interventions. However, studies investigating the determinants of *financial* outcomes of prevention related to implementation process are very seldom in the literature.

### Comparisons with the available literature

Only one review of economic evaluation studies focused on ergonomic interventions has been identified in the literature [16]. This review concludes that the level of evidence of financial merits of ergonomic interventions depends on the sector of activity: strong evidence was found in the manufacturing and warehousing sectors, moderate evidence in the administrative support and health care sectors and limited evidence in the transportation sector. Compared to this review, we included and analyzed eight additional studies together with their companion papers. Our review excluded several studies in Tompa et al.’s review because the scope of our review was slightly different: four studies were published before 2000, two were not focused on MSD, two evaluated only the costs or the effects of interventions and three involved interventions without an organizational dimension. Since our review focused on interventions with an organizational dimension, comparisons are limited because this has never been done in the literature.

In a broader perspective, reviews on economic evaluations of OHS preventive interventions in general are quite scarce in the literature. Hamberg et al. [26] conducted a systematic review on worksite mental health interventions and concluded that among 10 identified studies, the majority were of low methodological quality and the level of evidence did not make it possible to draw firm economic conclusions from the employer’s perspective. However, the authors emphasize that if we focus on the few high quality studies only, interventions aimed at preventing or treating mental health problems seem to be worth undertaking whereas return-to-work interventions do not. Tompa et al. [22] conducted a broad systematic review on OHS interventions and also underlined the small number of high quality studies available in the literature, which makes the level of evidence often insufficient. Nonetheless, they found strong evidence that multisector disability management interventions were worth undertaking. These results from systematic reviews of economic evaluations of preventive interventions are convergent with ours in that we also found a small number of high quality studies available in the literature and the level of evidence is often limited. However, the number of studies that establish an association between investment in prevention and positive financial outcomes for employers seem to be in the majority.

### Specific difficulties in the evaluation of ergonomic interventions and the need to improve quality in economic evaluation studies

The quality level of the included studies was low given that five studies out of nine had a low to very low score.

Some quality criteria are difficult to fulfill simultaneously. Indeed, it may be difficult to have both paired data and a low loss to follow-up depending on the turnover rate in organizations. In Québec, for instance, where the average yearly turnover rate was 33% in 2011 [70], it is difficult to reach simultaneously a loss to follow-up below 30% with paired data (criterion 9) and a length of follow-up after the end of intervention (criterion 10) longer than 3 months.

No mid-quality study (with a score between 45 and 60%) was found in our review. This can be explained by the fact that several criteria are necessarily interconnected. For example, the presence of a control group (criteria 4 and 5) is related to a comparative approach of competing alternatives and to an incremental analysis of costs and consequences (criterion 14). Therefore, the absence of a control group had a major impact on the total quality score. In economic evaluations, the inclusion of a control group is a crucial point since it makes it possible to estimate an incremental cost-effectiveness ratio or a net benefit based on a comparative approach between the intervention under study and a *do-nothing* alternative [20]. However, it may be very difficult to satisfy this requirement given the obstacles involved in data collection in workplaces and the need to define a control group comparable to the intervention group in terms of demographic features, work activity and organizational constraints, but with no contamination with the intervention group [71]. In our review, the five studies on lifting equipment installation show that this intervention leads to a total investment that is lower than the savings made after a payback period. However, none of these studies included a control group, thus resulting in a low quality score and a limited level of evidence. Our recommendation for improving the quality level of economic evaluations of ergonomic interventions would be to develop approaches with control groups in order to provide data for a comparative analysis, as is required for economic evaluations.

### Implementation process as an important tool for understanding economic results

Knowing more about the characteristics of the implementation process in the organization under study, such as support from supervisors, commitment of employees, pre-intervention analysis of workers’ needs and dose delivered, can help reproduce the keys of success in another organization or make it possible to avoid the main pitfalls [71]. This information is very relevant for decision-making given the lack of information on the generalizability of results from one organization to another. It is also consistent with Goldenhar et al. [72] and Kristensen [19] according to whom information about the implementation process is necessary in order to understand the effects of an intervention. For example, in the case of Bernaards’ study, the companion papers underline the fact that the WSPA results do not show that physical activity had an impact on WMSD pain, but rather that group meetings did not succeed in changing behaviors toward physical activity. This is a totally different way of interpreting the results of the economic evaluation.

In this context, the qualitative information provided by companion papers for our synthesis of the implementation process was very valuable in explaining some results found in the quantitative synthesis. However, the implementation process analysis remained exploratory and limited since information about implementation was very scarce and only two economic studies had companion papers that focused on the evaluation of the implementation process of the intervention. In the other companion papers, information was sometimes available in the discussion section, but it did not allow documenting all factors identified in our data extraction tool for implementation process. For example, information about the reach and satisfaction of targeted population, the fidelity to intervention protocol was very seldom. Moreover, it was difficult to distinguish between the data collected regarding implementation and the authors’ perception of the reasons for success or failure.

### Limitations and strengths

Some limitations have to be pointed out. We excluded studies that did not evaluate outcomes on WMSD exposure, symptoms or disorders. Some economic evaluation studies focused on a participatory ergonomic intervention but did not use WMSD status as a separate outcome. For example, it was impossible to disentangle effects on injuries of all kinds (including cuts and falls) from musculoskeletal-specific injuries [73, 74]. We also did not search the grey literature and thus did not include studies taken from reports or working papers not published through a peer-review process.

Our review also has several strengths. We followed a systematic procedure, with a large number of databases, a rigorous selection procedure with two independent evaluators for screening and quality assessment. Our review focused on interventions with an organizational dimension, which are of particular interest given the fact that they may be more difficult to implement than interventions that focus on the individual level only. We integrated qualitative and quantitative data extracted from economic evaluation and companion papers on the same intervention in order to better interpret economic evaluation results, an approach that is very rare in reviews of cost-benefit analysis of preventive interventions.

## Conclusion

We recommend that economic evaluations of preventive interventions should include information on the implementation process in order to facilitate the interpretation of economic evaluation results and enhance the generalizability of these results. This is also necessary for knowledge transfer and better utilization of research results for prevention-oriented decision-making in OHS. Employers wishing to invest in preventive interventions should recognize the importance of top and middle management support for and commitment to the intervention, employee’ participation, adequacy of intervention with workers’ needs as pre-requisites for improved musculoskeletal outcomes and positive financial outcomes.

## Additional file

Additional file 1:
**Table S1:** Economic results and quality assessment scores of the nine included studies: design, population, sample size, type of evaluation, economic perspective, indicators for outcomes and intervention costs, statistical analysis and results. **Table S2:** Intervention components and implementation data: description of the intervention and treatment of the control group, assessment of workers’ needs (diagnosis) before implementation and adequacy of intervention to workers’ needs, proportion of employees reached by the intervention, dose delivered and dose received, fidelity to intervention protocol, contextual factors and co-interventions or other confounders that may have had an impact on outcomes measures, obstacles and facilitators, authors’ hypotheses that may explain intervention effectiveness. (PDF 314 kb)

## Abbreviations

OHS: Occupational health and safety
WMSD: Work-related musculoskeletal disorders
WS: Work style
WSPA: work style and physical activity
