# Supplementary material for: Economic evaluations of ergonomic interventions preventing work-related musculoskeletal disorders: a systematic review of organizational-level interventions
Source: BMC Public Health. 2017 Dec 8;17:935. doi: 10.1186/s12889-017-4935-y (PMC5721617; doi:10.1186/s12889-017-4935-y)
Supplement: Additional file 1: — Table S1: Economic results and quality assessment scores of the nine included studies: design, population, sample size, type of evaluation, economic perspective, indicators for outcomes and intervention costs, statistical analysis and results. Table S2: Intervention components and implementation data: description of the intervention and treatment of the control group, assessment of workers’ needs (diagnosis) before implementation and adequacy of intervention to workers’ needs, proportion of employees reached by the intervention, dose delivered and dose received, fidelity to intervention protocol, contextual factors and co-interventions or other confounders that may have had an impact on outcomes measures, obstacles and facilitators, authors’ hypotheses that may explain intervention effectiveness. (PDF 314 kb) [file 12889_2017_4935_MOESM1_ESM.pdf]

Economic evaluations of ergonomic interventions preventing work-related musculoskeletal disorders: a systematic literature review of interventions with an organizational dimension

Hélène Sultan-Taïeb, Annick Parent-Lamarche, Aurélie Gaillard, Susan Stock, Nektaria Nicolakakis, Quan Nha Hong, Michel Vezina, Youssouph Coulibaly, Nicole Vézina, Diane Berthelette

Correspondance : Sultan_taieb.helene@uqam,ca

Appendix 1: Supplementary Tables S1 and S2

Table S1: Economic results and quality assessment scores of the nine included studies (principal economic article)

1st author, year of publication Country Objectives of intervention Quality assessment %	*Population, Total sample (experimental + control)*	*Design Type of economic evaluation Perspective*	*Outcomes indicators*	*Intervention costs indicators*	*Statistical analysis for the economic evaluation*	*Outcomes results, economic results*
Bernaards, 2011 [1] The Netherlands To reduce neck and upper limb symptoms and pain in computer workers. RSI@Work study *89%*	Computer workers with neck and upper limb symptoms in 7 companies. 3 groups: Work style plus physical activity (WSPA n=156) Work style intervention (WS n=152), usual care group (n=158).	RCT before-after. Paired data. Cost-effectiveness. Employer's perspective.	Average number of absence; frequency of absenteeism periods, 12-month prevalence of absenteeism, long-term absenteeism, short-term absenteeism Pain intensity, recovery.	Costs of the counsellors: training, time spent at group meetings. Costs of elastic bands used in the WSPA group. Costs of breaks and exercise reminder software. Cost due to absenteeism because of time spent by workers at meetings.	Linear regression analysis to adjust for pain at baseline. Bootstrap. Acceptability curves. Sensitivity analyses with elasticity values of production losses and with or without data imputation.	Differences in costs between groups not significant. WS intervention was cost-effective in reducing average pain and improving recovery in neck/shoulder but not recovery in arm/wrist/hand. WSPA was not more effective than usual care.
Collins, 2004 [2] United States To prevent back injuries due to resident lifting among nurses. *37%*	All nursing staff (n=1728), company records.	Quasi-experimental (not randomised) before-after. Control group data are not used in the economic analysis. Unpaired data Pay-back period. Employer's perspective.	3 data sources for injury data: Workers' compensation injury claims data, OSHA injury data, first reports of employee injury.	Costs of equipment, employee training.	Difference between costs and benefits with no comparison with an alternative strategy.	The total investment is lower than the savings cumulated after three years (payback period <3 years), the approach is not incremental.
de Jong, 2002 [3] The Netherlands To reduce MS workload in installation work. *29%*	Employees of a large installation company (n=7000). No information on the number of employees who participated in the process.	Quasi-experimental not randomized uncontrolled before-after. Unpaired data. Payback period. Employer's perspective.	Daily hours of lifting/carrying, of trunk flexion, of kneeled posture converted into monetary units.	Costs of time spent on training and meetings. Costs of equipment. No information on calculation methods and data sources.	Cost-effectiveness ratio based on a limited data collection (approx. 10 qualitative interviews). No comparison with an alternative strategy.	Users reported a reduction in manual lifting/carrying but not kneeled work. The payback period is less than 1 year, the approach is not incremental.
Driessen, 2012 [4] The Netherlands To prevent low back and neck pain. Stay@work participatory ergonomic (PE) program. *69%*	4 Dutch companies: railway transportation, airline, university and steel company. Intervention group (n=1472) with PE program. Control group (n=1575) without PE program.	RCT before-after. Paired data. Cost-effectiveness from societal perspective. Cost-benefit analysis from employers' perspective.	Cost-effectiveness: Prevalence of LBP and NP. Cost benefit: Health care costs (costs of medical procedures, medication). Cost of sick leave (friction cost approach).	Cost of study protocol development, cost of training, and cost of ergonomists time. Costs of time spent by workers. Costs of room rental.	Multiple imputations (intention-to-treat principle). Bootstrap. Incremental cost effectiveness ratio. Difference between costs and benefits.	Difference in costs not significant between the two groups. Differences in effects not significant. From a societal perspective, PE was not cost-effective compared to control for LBP and NP prevalence, work performance, and sick leave. CBA from a company perspective showed a loss of 78 Euros/worker.

1st author, year of publication *Country* *Objectives of intervention* *Quality assessment %*	*Population, Total sample (experimental + control)*	*Design* *Type of economic evaluation* *Perspective*	*Outcomes indicators*	*Intervention costs indicators*	*Statistical analysis for the economic evaluation*	*Outcomes results, economic results*
Engst, 2005 [5]<br>Canada<br>To reduce the risk of MS injuries due to resident lifting among healthcare workers.<br>*29%*	Healthcare workers, no information on sample size (data source: enterprise records)	RCT before-after (but control group not included in calculation).<br>Paired data.<br>Payback period.<br>Perspective of workers' compensation board of British Columbia.	Claims costs for MS injuries. Other savings may have been included but are not documented in the article.	No information on assessment method.	Pre-post difference for costs and savings.<br>Two-way repeated measures ANOVA with matched sample for workers perception.	Payback period estimated at 9.6 years for all handling claims and at 6.5 years for lift and transfer claims only.<br>Information available in the article does not allow validation of payback period calculation.
Nelson, 2006 [6]<br>United states<br>To reduce MSD injuries due to patient handling among nurses.<br>*43%*	Nursing staff (n=300)	Quasi-experimental uncontrolled randomized before-after.<br>Paired data.<br>Payback period.<br>Employer's perspective.	Effectiveness:<br>Injury rate, lost work days, job satisfaction, self-report unsafe handling practices<br>Payback period:<br>Cost of medical treatment; workers' compensation costs; cost of lost productivity: sick days, modified days (valued as half of wages and benefits).	Cost of device, cost of maintenance and installation, nursing staff training costs.	Poisson regression model for pre-post differences in injury rate. Paired t-tests.<br>Pre-post difference for costs and savings.<br>No comparison with an alternative strategy.	Decrease in the rate of injuries, in number of modified duty days after injury, in number of unsafe handling practices, increase in job satisfaction.<br>Payback period of 3.75 years (if savings remain the same after the first year). The approach is not incremental.
Oude Hengel, 2014 [7]<br>The Netherlands<br>To improve work ability, physical and mental health and reduce MSD symptoms among construction workers.<br>*83%*	Construction workers from 6 companies (n=293).<br>Intervention group (n=171) received the prevention program.<br>Control group (n=122) did not receive the program.	RCT before-after.<br>Paired data.<br>Cost-effectiveness.<br>Cost-benefit and ROI.<br>Employer's perspective.	Cost effectiveness:<br>Work ability,<br>Physical and mental health,<br>Prevalence of MS symptoms,<br>Cost-benefit:<br>Productivity avoided costs (sickness absenteeism, presenteeism)	Costs of training sessions, material costs.<br>Costs of paid time of workers to participate were not included since control group had other training sessions of the same duration.<br>Avoided absenteeism costs are subtracted from intervention costs in cost-effectiveness analysis.	Multiple imputation (intention-to-treat principle).<br>Regression analyses to adjust for baseline values between the two groups.<br>Bootstrap.<br>Mean differences with confidence intervals.3 sensitivity analysis: inclusion of presenteeism costs, complete cases only, participation in all sessions only.	Cost-effectiveness: total costs were lower in intervention group. Outcomes differences were not significant between the two groups. Therefore, the intervention is not cost-effective.<br>Cost-benefit (avoided absenteeism costs – intervention costs): positive net benefit per worker of 641 euros, with a ROI of 544%.

1st author, year of publication<br>Country<br>Objectives of intervention<br>Quality assessment %	Population,<br>Total sample (experimental + control)	Design<br>Type of economic evaluation<br>Perspective	Outcomes indicators	Intervention costs indicators	Statistical analysis for the economic evaluation	Outcomes results, economic results
Sedlak, 2009 [8]<br>United States<br>To reduce MS injury due to residents lifting and handling.<br>*37%*	Healthcare workers (n=52)	Quasi-experimental randomized uncontrolled before-after.<br>Paired data.<br>Payback period.<br>Employer's perspective.	Effectiveness:<br>Incident reports<br>Perceptions of injuries<br>Payback period:<br>Compensation records	Equipment cost	All data were analyzed using descriptive statistics showing change scores in percentages of reduction of the dependant variables. No inferential statistics. No comparison with an alternative strategy.	Reduction in workers' compensation claims, paid claims related to lifting, perception of injuries, modified work days.<br>Payback period of approximately 5 years. The approach is not incremental.
Spiegel 2002 [9], completed by Chhokar 2005 [10]<br>Canada<br>To reduce the risk of MS injuries due to resident lifting among healthcare workers.<br>*63%*	Healthcare workers, no information on sample size (data source: enterprise records)	Quasi-experimental uncontrolled before-after.<br>Unpaired data.<br>Payback period, ROI.<br>Perspective of workers' compensation board (WCB) of British Columbia for payback period and ROI (Spiegel 2002, Chokkar, 2005).<br>Perspective of the employer for ROI (Spiegel 2002).	Claims costs for MS injuries.<br>Costs savings comparison between observed claims costs after intervention and a simulation of claims costs under assumption of no intervention.<br>Indirect cost savings for the employer assumed to be twice the amount of direct costs.	Installation costs of ceiling lifts, operating costs, cost of redeployed equipment.	The difference between two trends of claims costs is measured (observed trend post intervention and simulated trend without intervention).<br>Spiegel 2002: simulation based on two assumptions (stable or increasing)<br>Chhokar 2005: Linear regression and t-tests produce two simulated trend slopes.	From WCB perspective: depending on simulation methods: Intervention costs 344 323$ paid back within 2 to 3.85 years (Spiegel 2002) or within 0.82 to 2.5 years (Chhokar 2005). Annual ROI 8.1% (Spiegel 2002).<br>From employer's perspective: discounted value of savings for 12 years (lift life span) gives an annual ROI from 6.2 to 22.9% depending on simulation methods (Spiegel 2002).

CBA: Cost-benefit analysis; LBP: Low back pain; MS: Musculoskeletal; MSD: Musculoskeletal disorders; NP: Neck pain; OSHA: Occupational safety and health administration; PE: Participatory ergonomics; RCT: Randomized-controlled trial; ROI: Return on investment rate; WCB: Workers' compensation board; WS: work style; WSPA: work style and physical activity.

Table S2: Intervention components and implementation data (principal economic article and companion papers)

Reference Companion papers (CP)	*Intervention description*	*Intervention duration (i) Follow up after end of intervention (f)*	*Control group treatment*	*Needs assessment and adequacy of intervention to workers' needs*	*Dose delivered (DD), Dose received (DR), Reach of target pop (R), Protocol Fidelity (F)*	*Co-interventions and contextual factors*	*Identification of obstacles and facilitators (OF) Authors' hypothesis for intervention ineffectiveness (H), Satisfaction (S)*
Bernaards 2011 [1] CP: Bernaards 2006 [11] 2007 [12] 2008 [13]	Six interactive group meetings at the workplace, during work time under supervision of a specially trained counsellor. Goal: behavioral change toward work style (Work style group) and Physical activity (Work style plus Physical activity group). Work style refers to body posture, static workload, insufficient breaks, high workload, work stress. Physical activity component consisted in engagement toward physical activity. Steps: to provide information and raise awareness, to discuss and find solutions for barriers regarding behavioral change.	6 months (i) 6 months (end of intervention) and 12 months (f)	Usual care : no participation to group meetings, possible care by occupational physician	Pilot study results: Participants expected bad workstation setup to be the primary cause of symptoms. They did not think that the main cause was a lack of physical activity (- for WSPA)	DR: Attendance to three meetings was similar for WS and WSPA groups (82 % and 72%) (+). But attendance to five meetings was higher for WS group (40.1%, 28.8% for WSPA) (- for WSPA).	Workers who were under treatment of a doctor were excluded to avoid bias. Among participants, those who wanted to visit a doctor or take medications had to report it. Absenteeism variations might be due to reorganisation in one company.	H: The combination of improving work style and increasing physical activity behaviour may have caused a lack of focus resulting in smaller behavioural changes (- for WSPA).<br>H: Group meeting may not be suitable for increasing physical activity, which may explain a larger dropout rate in WSPA group (- for WSPA).
Collins 2004 [2] no CP	<u>Equipment:</u> Resident handling equipment for repositioning (friction reducing sheets) and transfering (full body lift or stand-up lift)<br><u>Written zero lift policy:</u> Written guidelines for assessing each resident's transferring needs and procedures.<br><u>Training to all staff:</u> 30 min knowledge-based training and demonstration + 45 min with job specific content according to residents' needs.	6 years (i) 3 years (f)	No control group in the economic analysis.	No information available	No information available	Medical care provided to nurses remained the same before, during and after intervention.	OF: Nurses could evaluate and provide input on the selection of lifting equipment, which favored staff participation and buy-in (+).
de Jong et Vink, 2002 [3] no CP	Participatory approach in 6 steps: preparation, problems analysis, selection of solutions according to problem prioritization, prototyping and test, implementation, evaluation. Three solutions were implemented: aid to transport switch cupboards, raiser for bending pipes, assemble seat for floor work.	Intervention duration not available. Baseline-follow-up duration: 18 months.	No control group.	7 types of work were identified by the steering group supported by business units. 3 types of activities appeared to cause problem. Problems were	DD: 3 major solutions were implemented out of 9. The other 6 solutions were made available and marginally adapted (+).<br>DR: 8 out of 10 business units	No information available	OF: Top management showed a great commitment to and support for this intervention (+).<br>OF: Differences in work activities between units and differences in applicability (-).<br>OF: Limited acceptance by employees in some units (-)

Reference Companion papers (CP)	*Intervention description*	*Intervention duration (i) Follow up after end of intervention (f)*	*Control group treatment*	*Needs assessment and adequacy of intervention to workers' needs*	*Dose delivered (DD), Dose received (DR), Reach of target pop (R), Protocol Fidelity (F)*	*Co-interventions and contextual factors*	*Identification of obstacles and facilitators (OF) Authors' hypothesis for intervention ineffectiveness (H), Satisfaction (S)*
				prioritized based on questionnaires. 9 solutions were developed with the participation of employees, supervisors, the steering group (+).	adopted at least 1 of the 9 solutions. 4 business units used them daily (+).		H: Adding organizational measures or system solutions and more direct participation could have increased the impact (-).
Driessen 2012 [4] CP: Driessen 2008 [14] 2010a[15] 2010b[16] 2011a[17] 2011b[18]	A trained ergonomist guided a working group (8 workers and 1 manager) in each department to evaluate and prioritise risk factors for low back and neck pain, elaborate and implement solutions. A six-hour meeting plus a four-hour implementation training. Solutions had to be implemented within 3 months. 66 ergonomic measures were prioritized by working groups, among which 34% were implemented.	Intervention duration not clear. Repeated measures at baseline, three-, six-, nine, and 12-month follow-up.	The control group did not have participatory ergonomic program, watched 3 short films as a sham intervention.	Actions were the result of an analysis of risk factors and elaboration of solutions by working groups. But the prevalence of LBP and NP was very low at baseline (-).	DD: 34% of prioritized ergonomic measures perceived as fully implemented by implementers (-). DR: 26% of workers perceived ergonomic measures as fully implemented (-). F: High attendance of steering groups, validation of the steps and quality of training (+)	Some ergonomic co-interventions occurred in both intervention and control groups but none of them were LBP or NP prevention interventions.	OF: Lack of financial and personnel resources (-). H: working groups prioritized the most simple and less expensive measures, which may explain absence of effects (-). Loss-to-follow up was very high maybe because more than 70% workers did not have LBP or NP. S: High levels of satisfaction among working group members (+) and lower among workers (-).
Engst, 2005 [5] no CP	Installation of ceiling lifts for 75 beds in a healthcare unit. 1-h training session provided to all staff. No-unsafe manual lift policy signed by employers and unions.	6 months (i) 21 months (f)	No ceiling lifts in a similar 75-bed healthcare unit.	Based on the literature (ceiling lifts in healthcare sector). Resident needs may have changed between pre- and post-intervention (-).	F: Equipment installation confirmed (+) (no information on training nor no-unsafe lift policy)	No information available	OF: Ceiling lifts were not used for repositioning patients because it required more time (- for repositioning). H: Ceiling lifts seemed to better meet workers' needs for lifting and transferring than repositioning (- repositioning).
Nelson 2006 [6] CP: Nelson, 2003 [19]	Ergonomic program based on risk assessment protocol, with a patient handling assessment, algorithms to support handling decisions, installation of equipment, after	1 month (i) 9 months (f)	No control group.	The assessment protocol included collecting data to identify high-	No information available	Very high staff turnover (65%), nurses going from one unit to another with	OF: Strong support from nurse managers for the program, strong co-workers support and patients became more

Reference Companion papers (CP)	*Intervention description*	*Intervention duration (i) Follow up after end of intervention (f)*	*Control group treatment*	*Needs assessment and adequacy of intervention to workers' needs*	*Dose delivered (DD), Dose received (DR), Reach of target pop (R), Protocol Fidelity (F)*	*Co-interventions and contextual factors*	*Identification of obstacles and facilitators (OF) Authors' hypothesis for intervention ineffectiveness (H), Satisfaction (S)*
	action reviews, a no-lift policy was signed with the hospital management.			risk units, obtaining feedback from nurses, site visits (+).		different work environments.	supportive after implementation (+).<br>H: Duration of intervention was short (1 month) before collecting data (-).
Oude Hengel 2014 [7] CP: Oude Hengel 2010 [20] 2011 [21] 2012 [22]	Individual level, physical component: A first 30-minute training sessions with a physical therapist based on a 15-minute observation of the worksite. 3 individual recommendations on how to reduce physical workload. A second 30-min session (4 months later) for advice follow-up.<br>Rest-break tool to fill in each week in order to raise awareness.<br>Group level, mental component: two empowerment sessions in order to improve workers' influence on work, including how to communicate with supervisor.	6 months (i)<br>Baseline to follow-up: 3, 6, 12 months.<br>6 months (f)	Traditional training sessions about physical workload, safety issues, learning new materials.	No information available.	DD: Almost all worksites received the training sessions (90-100%) (+)<br>DR: 39% of workers followed 1 or 2 sessions out of 4 sessions (-)<br>F: Supervisors and management did not participate in empowerment sessions, physical sessions were not all accomplished, only 44% workers filled in the Rest-break tool weekly (-).	Other factors such as improved job control of less manual handling might have contributed to reduction of absenteeism. Economic crisis climate and increased job insecurity (-).	H: Signs of program failure in the intervention because of low dose received and fidelity (-).<br>S: Workers considered it difficult to fill in the Rest-break tool. Low satisfaction towards empowerment sessions which did not involve supervisors (-).
Sedlak 2009 [8] no CP	Equipment (ceiling lifts, sit-to-stand devices, and fast-rising beds).<br>Education program to all healthcare staff regarding safe patient-handling principles and case scenarios illustrating lifting protocols and selection of appropriate equipment for a variety of situations.	7 months (i)<br>6 months (f)	No control group	Assessment of workers' perceptions of injuries related to lifting and transferring (+).	No information available	No information available	No information available
Spiegel, 2002 [9] CP : Chhokar 2005 [10] Ronald 2002 [23]	Installation of 65 ceiling lifts.<br>Training on patient handling with ceiling lifts was provided to all staff.<br>No-unsafe manual lift policy.	Spiegel :<br>5 months (i)<br>12 month(f)<br>Chhokkar:<br>3 years (f)	No group control. Effects without intervention are simulated.	Based on the literature (ceiling lifts in healthcare sector)	F: Equipment installation confirmed, ad hoc training conducted by the supplier as needed (+). No information on no-unsafe manual lift policy	Several potential factors (not evaluated): changes in staffing ratios, job stress, regional changes in compensation legislation during follow-up (-).	OF: Injuries after intervention mainly due to resisting or heavy patients, patient who slipped, and procedural error by caregiver (-).<br>H: Potential problems in the use of ceiling lifts for repositioning tasks (-).

CP: Companion paper; LBP: Low back pain; NP: Neck pain; "+" indicates a feature perceived as favorable to intervention success, "-" perceived as contributed to intervention failure by the authors.

References (economic evaluation article and companion papers)

1. Bernaards CM, Bosmans JE, Hildebrandt VH, van Tulder MW, Heymans MW: The cost-effectiveness of a lifestyle physical activity intervention in addition to a work style intervention on recovery from neck and upper limb symptoms and pain reduction in computer workers. Occup Environ Med 2011, 68(4):265-272.
2. Collins JW, Wolf L, Bell J, Evanoff B: An evaluation of a "best practices" musculoskeletal injury prevention program in nursing homes. Injury prevention : journal of the International Society for Child and Adolescent Injury Prevention 2004, 10(4):206-211.
3. de Jong AM, Vink P: Participatory ergonomics applied in installation work. Applied ergonomics 2002, 33(5):439-448.
4. Driessen M, Bosmans J, Proper K, Anema J, Bongers P, van der Beek A: The economic evaluation of a participatory ergonomics programme to prevent low back and neck pain. Work 2012, 41 Suppl 1:2315-2320.
5. Engst C, Chhokar R, Miller A, Tate R, Yassi A: Effectiveness of overhead lifting devices in reducing the risk of injury to care staff in extended care facilities. Ergonomics 2005, 48(2):187-199.
6. Nelson A, Matz M, Chen F, Siddharthan K, Lloyd J, Fragala G: Development and evaluation of a multifaceted ergonomics program to prevent injuries associated with patient handling tasks. International journal of nursing studies 2006, 43(6):717-733.
7. Oude Hengel K, Bosmans JE, Van Dongen JM, Bongers PM, Van der Beek AJ, Blatter BM: Prevention program at construction worksites aimed at improving health and work ability is cost-saving to the employer: Results from an RCT. American journal of industrial medicine 2014, 57(1):56-68.
8. Sedlak CA, Doheny MO, Jones SL, Lavelle C: The clinical nurse specialist as change agent: reducing employee injury and related costs. Clinical nurse specialist CNS 2009, 23(6):309-313; quiz 314-305.
9. Spiegel J, Yassi A, Ronald LA, Tate RB, Hacking P, Colby T: Implementing a resident lifting system in an extended care hospital. Demonstrating cost-benefit. Am Assoc Occup Health Nurses Journal 2002, 50(3):128-134.
10. Chhokar R, Engst C, Miller A, Robinson D, Tate RB, Yassi A: The three-year economic benefits of a ceiling lift intervention aimed to reduce healthcare worker injuries. Applied ergonomics 2005, 36(2):223-229.
11. Bernaards CM, Ariëns GA, Hildebrandt VH: The (cost-) effectiveness of a lifestyle physical activity intervention in addition to a work style intervention on the recovery from neck and upper limb symptoms in computer workers. BMC musculoskeletal disorders 2006, 7(1):1.
12. Bernaards CM, Ariens GA, Knol DL, Hildebrandt VH: The effectiveness of a work style intervention and a lifestyle physical activity intervention on the recovery from neck and upper limb symptoms in computer workers. Pain 2007, 132(1-2):142-153.
13. Bernaards CM, Ariens GA, Simons M, Knol DL, Hildebrandt VH: Improving work style behavior in computer workers with neck and upper limb symptoms. Journal of occupational rehabilitation 2008, 18(1):87-101.
14. Driessen MT, Anema JR, Proper KI, Bongers PM, van der Beek AJ: Stay@ Work: Participatory Ergonomics to prevent low back and neck pain among workers: design of a randomised controlled trial to evaluate the (cost-) effectiveness. BMC Musculoskeletal Disorders 2008, 9(1):1.

15. Driessen MT, Proper KI, Anema JR, Bongers PM, van der Beek AJ: Process evaluation of a participatory ergonomics programme to prevent low back pain and neck pain among workers. Implementation science : IS 2010, 5:65.
16. Driessen MT, Groenewoud K, Proper KI, Anema JR, Bongers PM, van der Beek AJ: What are possible barriers and facilitators to implementation of a Participatory Ergonomics programme? Implementation science : IS 2010, 5:64.
17. Driessen MT, Proper KI, Anema JR, Knol DL, Bongers PM, van der Beek AJ: The effectiveness of participatory ergonomics to prevent low-back and neck pain--results of a cluster randomized controlled trial. Scandinavian journal of work, environment & health 2011, 37(5):383-393.
18. Driessen MT, Proper KI, Anema JR, Knol DL, Bongers PM, van der Beek AJ: Participatory ergonomics to reduce exposure to psychosocial and physical risk factors for low back pain and neck pain: results of a cluster randomised controlled trial. Occup Environ Med 2011, 68(9):674-681.
19. Nelson A, Owen B, Lloyd JD, Fragala G, Matz MW, Amato M, Bowers J, Moss-Cureton S, Ramsey G, Lentz K: Safe Patient Handling and Movement: Preventing back injury among nurses requires careful selection of the safest equipment and techniques. The second of two articles. AJN The American Journal of Nursing 2003, 103(3):32-43.
20. Oude Hengel KM, Joling CI, Proper KI, Blatter BM, Bongers PM: A worksite prevention program for construction workers: design of a randomized controlled trial. BMC public health 2010, 10(1):1.
21. Oude Hengel KM, Blatter BM, van der Molen HF, Joling CI, Proper KI, Bongers PM, van der Beek AJ: Meeting the challenges of implementing an intervention to promote work ability and health-related quality of life at construction worksites: a process evaluation. Journal of occupational and environmental medicine / American College of Occupational and Environmental Medicine 2011, 53(12):1483-1491.
22. Oude Hengel KM, Blatter BM, Joling CI, van der Beek AJ, Bongers PM: Effectiveness of an intervention at construction worksites on work engagement, social support, physical workload, and need for recovery: results from a cluster randomized controlled trial. BMC Public Health 2012, 12(1):1008.
23. Ronald LA, Yassi A, Spiegel J, Tate RB, Tait D, Mozel MR: Effectiveness of installing overhead ceiling lifts. Reducing musculoskeletal injuries in an extended care hospital unit. AAOHN journal : official journal of the American Association of Occupational Health Nurses 2002, 50(3):120-127.
